# Characteristics of 10-Methacryloyloxidecyl Dihydrogen Phosphate Monomer in Self-Etching Two-Bottled Dental Adhesive System: Comparison with Commercial Products

**DOI:** 10.3390/ma13163553

**Published:** 2020-08-12

**Authors:** Jiyeon Roh, Hyunjung Shin, Min-Ho Hong

**Affiliations:** 1 Research Planning and Coordination Division, National Forensic Service, Wonju-si 26460, Korea; hindhorn@gmail.com; 2 Nature Inspired Materials Processing Research Center, Department of Energy Science, Sungkyunkwan University, Suwon-si 16419, Korea; hshin@skku.edu

**Keywords:** self-etching two bottled dental adhesive, 10-methacryloyloxidecyl dihydrogen phosphate monomer, biocompatibility, shear bond strength

## Abstract

Dentin bonding is a key in restorative dentistry. Here, we developed a self-etching two-bottle adhesive system containing 10-methacryloyloxidecyl dihydrogen phosphate monomer (MDP) and the physical, mechanical, and biocompatible properties were evaluated. The characteristics of MDP were analyzed using nuclear magnetic resonance (NMR). Tests for water sorption and solubility, the shear-bond strengths to dentin and enamel, and cytotoxicity were performed. The newly-blended experimental group showed the lowest thickness and water sorption and solubility values. The shear bond strength of enamel and dentin were comparable to control groups (the three other products were Clearfil^TM^, UniFil^®^, and AdheSE^®^). All test groups showed 60% of cell viability. In this study, the properties of the newly-synthesized adhesive are comparable with the others. The fundamental goal of this study is to get the MDP patent released, as it is intended for domestic production. For this purpose, this dentin adhesive was developed and compared with the commercial product.

## 1. Introduction

The dental adhesive market has been increasing over the last half century, with dentin adhesives being developed along with other restorative materials in dentistry. Many new adhesives have been proposed in recent times. With better understanding of the smear layer and composition of dentin, the formulation of dentine adhesives has changed over time [1]. At the time of using the dental adhesive of the no-etch system, all adhesives were required to have total etching such as a phosphoric acid for improvement of bond strength. After that, when using the total-etch system, the wet bonding system was introduced, and enamel and dentin were treated differently [2]. The acidic functional self-etching monomer was also introduced, and self-etching adhesives were developed. As such, the etching system has shown various paradigms, and as a result, the etching system has been developed in various ways.

Nowadays, acid functional monomer 10-methacryloyloxidecyl dihydrogen phosphate monomer (MDP) can be considered as a gold-standard functional matter in any self-etching system in terms of chemical bonding and clinical longevity [3]. From its development, this monomer has required royalties to be paid due to the patent period [4]. However, after the patent period ended, synthesis and use of the monomer were allowed. Although dentin adhesives have improved tremendously over the past decade, the properties of the new generation have not been proven yet. In addition, its dependence on imports is high, and few companies and research centers focus on and invest in dental adhesives.

Among the various adhesives, self-etching adhesive systems have been widely used because of their shorter application time. Self-etching systems are generally less technique-sensitive compared to employing separate acid conditioning and rinsing steps [5]. In addition, the collapse of dentin collagen and the production of demineralized collagen are prevented. Despite the presence of short resin tags and intact smear plugs, a good seal has been reported. Kijasamanmith et al. reported that there was an improvement in the regional tensile strength test over the pulp horn regions when compared with systems that utilize a total etch technique [6].

Several self-etching and self-priming adhesives have been introduced recently. Among the products, CLEARFIL^TM^ SE BOND (Kuraray, Okayama, Japan) is a multi-purpose system containing an unsaturated methacrylate phosphate ester and MDP as the acidic resin monomer. AdheSE^®^ (Ivoclar Vivadent, Schaan, Liechtenstein) is a two-bottle self-etching system which contains dimethacrylate phosphoric acid acrylate initiators and hydrophilic monomer. Moreover, UniFil^®^ (GC Corp., Tokyo, Japan) is also a two-bottle self-etching system with 4-MET and hydrophilic monomer [7].

In this study, we developed a two-bottle self-etching adhesive system containing MDP and hydrophilic monomer. In addition, the physical, mechanical, and biocompatible properties were evaluated. Commercial two-bottle products on the market were used as control groups. The null hypothesis of this study was that there were no significant differences between the experimental and control groups.

## 2. Materials and Methods

### 2.1. Materials

Using the monomer, the primer and bonding were blended, and brand-named products such as Nexo (Nexobio, Cheongju-si, Korea). Clearfil^TM^ (Kuraray, Okayama, Japan), UniFil^®^ (GC Corp., Tokyo, Japan), and AdheSE^®^ (Ivoclar Vivadent, Schaan, Liechtenstein) were purchased and used as control groups. L929 fibroblasts were obtained from the Korean Cell Line Bank (Seoul, Korea). Roswell Park Memorial Institute (RPMI) 1640 medium, fetal bovine serum (FBS), and 1% antibiotics/antimycotic solution (Penicillin/Streptomycin) were purchased from Gibco (Grand Island, NY, USA). In addition, 3-(4,5-dimethylthiazol-2-yl)-2,5-diphenyltetrazolum bromide (MTT; Sigma-Aldrich, San Diego, CA, USA) and dimethyl sulfoxide (DMSO; Amresco, Solon, OH, USA) were used.

### 2.2. Physical and Mechanical Properties

The characteristics of MDP were analyzed using ^1^H nuclear magnetic resonance (NMR; Avance II, Bruker Biospin, Rheinstetten, Germany). In short, the monomer was diluted in acetone. The accumulation and repetition times were 1000 times and 15 s, respectively.

The bonding agent (20 μg) was placed on the center of a glass plate and covered with another glass plate. Each glass had a contact surface area of 200 ± 25 mm^2^. With a loading device, the bonding agent was loaded (150 ± 2 N) on the glasses for 180 ± 10 s. Then, it was light cured for 20 s with 1200 mW/cm^2^ using a LED curing system (Elipar^TM^ S10; 3M ESPE, Seefeld, Germany) with reference to the description recommended by the manufacturer. The combined thickness of the two glass plates and the specimen film were recorded using a micrometer. The film thickness test was carried out five times.

The water sorption (W_sp_), solubility (W_sl_), and thickness were evaluated according to ISO 4049 [8]. The mold (*d* 15 ± 0.1 mm × *h* 1.0 ± 0.1 mm) was then filled with bonding agent and covered with a polyethylene film and slide glass. After removing the specimen from the mold, its volume (V) was measured. It was then weighed (m_1_), and the samples were placed in a 6-well plate and immersed in 10 mL of distilled water. The immersed samples were placed in a 37 °C water bath for 7 days, after which they were removed. The moisture on the surfaces was removed and measured (m_2_) and the specimens were placed in a new 6-well plate and stored in a desiccator maintained at room temperature. Each disk was weighed daily until a constant dry mass (m^3^) was obtained. Water sorption and solubility were calculated using the following equations.

W_sp_ (μg/mm^3^) = (m_2_ − m_3_)/V (1)

W_sl_ (μg/mm^3^) = (m_1_ − m_3_)/V (2)

The shear bond strength to enamel or dentin was tested according to ISO 29022 [9]. The embedded teeth in acrylic resin were polished with a 600-grit silicon-carbide (SiC) sandpaper (R&B, Daejeon, Korea) until the enamel or dentin surface was exposed. The primer was applied immediately after removing it from water after which the bonding agent was applied and light cured for 20 s with 1200 mW/cm^2^ according to the manufacturer’s protocol. The composite resin was condensed in the mold (*d* 2.38 ± 0.03 mm), and the sample composite button was placed and aligned to the notched blade. Loading was done with a universal tensile machine (Instron 5942, Instron, Norwood, MA, USA) with a crosshead speed of 1.0 ± 0.1 mm/min until failure [10]. The maximum force (N) prior to failure of the bond was recorded and the bond strength was calculated using the formula.

σ (MPa) = F (N)/A_b_ (mm^2^) (3)

### 2.3. In Vitro Cytotoxicity Test

The MTT assay was performed to evaluate the cytotoxicity according to ISO 10993-5 [11]. In brief, the specimens with 10 ± 0.1 mm in diameter and 1 ± 0.01 mm in height were prepared. The sample was sterilized under UV light for 30 min and immersed in serum-free media (3.0 cm^2^/mL) according to ISO 10993-12 [12]. After a day, the supernatant was decanted into another well and diluted with serum-free media. Mouse fibroblast cell line, L929, was seeded at 1 × 10^4^/cells in a 96-well plate, in 100 μL of cell culture medium RPMI 1640 with 10% of FBS. After a day, the medium was changed to extractions from each type of material (1:1) or dilutions of extractions with serum-free media (1:2). After another day, the extractions were removed and 50 μL of MTT solution (1 mg/mL) were added. After two hours, the solutions were changed to 100 μL of DMSO to dissolve purple formazan. After that, the microplates were read using a microplate reader (Epoch microplate spectrophotometer, Synergy-BIOTEK, Winooski, VT, USA). The tests were repeated three times and all results were calculated as relative values by dividing the negative control group.

### 2.4. Statistical Analysis

In all experiments (except a ^1^H NMR spectrum analysis), statistical analyses including one-way ANOVA tests were performed for all data using SPSS 23 statistical software (IBM, Armonk, NY, USA), and a post hoc analysis was performed using Tukey’s method. The significant difference between each group was determined at a *p*-value <0.05.

## 3. Results

**Figure 1:**
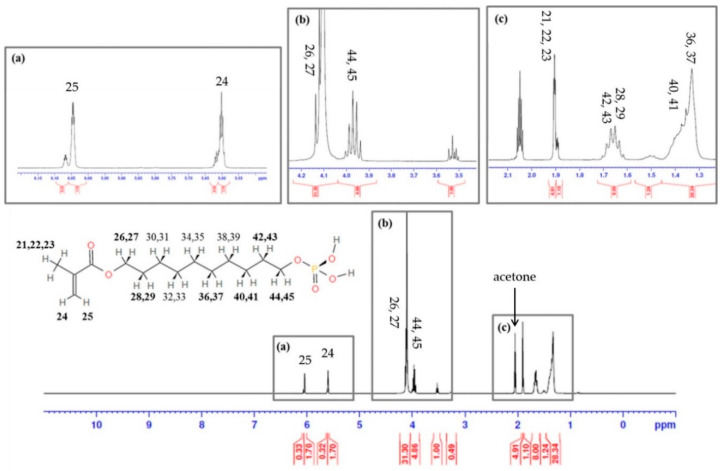
Confirmation of terminal double bond groups for polymerization of MDP (10-Methacryloyloxidecyl Dihydrogen Phosphate) by ^1^H NMR spectrum. (**a**) Polymerizable group; (**b**) hydrophobic group; (**c**) polymerizable and hydrophobic groups with acetone peak.

The monomer was dissolved in acetone and ^1^H NMR spectrum analysis was performed. There was an acetone peak in Figure 1c. Each hydrogen network was written in the chemical structure. The peak patterns were matched with the chemical structure of MDP. The boxes were labelled with alphabets and the upper boxes were magnified to clarify the peaks. The simplified molecular-input line-entry system (SMILE) and chemical abstracts service (CAS) number are described in Table 1.

**Figure 2:**
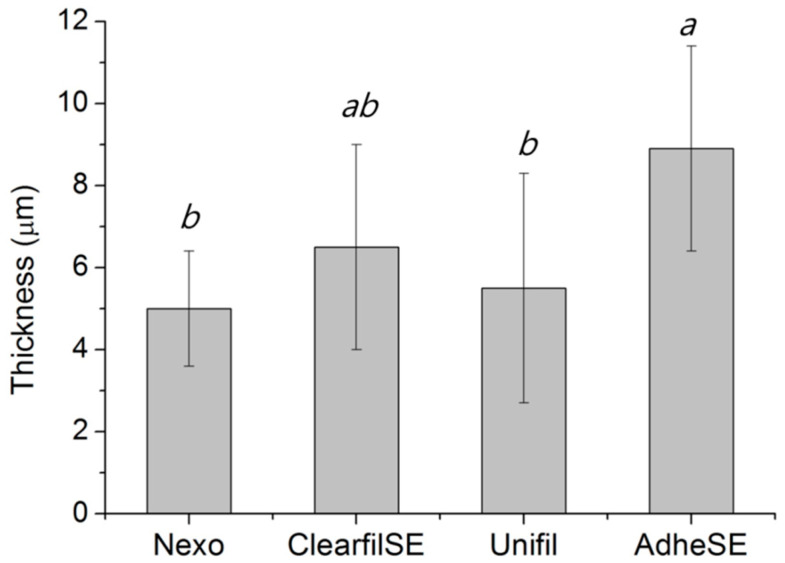
Thickness of bonding agent (μm). The same letters mean no significant differences. Raw data are presented in Table S1.

**Figure 3:**
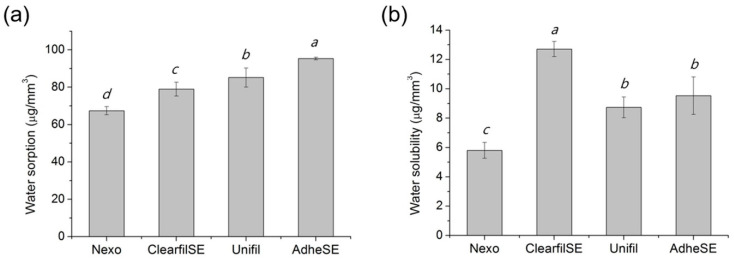
Water sorption (**a**) and solubility (**b**). The same letters mean no significant differences. Raw data are presented in Tables S2 and S3.

**Figure 4:**
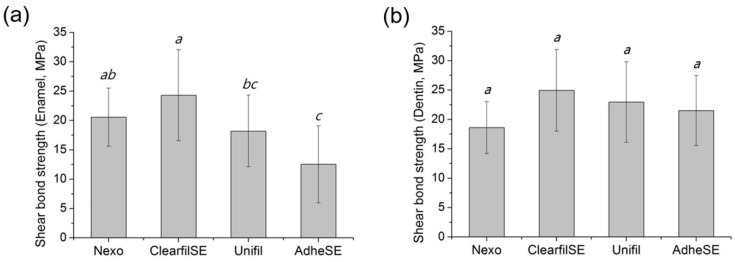
Shear-bond strength to enamel (**a**) and to dentin (**b**). The same letters mean no significant differences. Raw data are presented in Tables S4 and S5.

The film thickness was measured to confirm the validity for other tests. The results of film thickness are shown in Figure 2. In this test, the Nexo and Unifil groups showed the lowest thickness. The results of water sorption and solubility are shown in Figure 3. The requirements of ISO 4049 are below the value of 40 μg/mm^3^ in W_sp_ and 7.5 μg/mm^3^ in W_sl_ [8]. The AdheSE had the highest value in W_sp_ and Clearfil SE has the highest value in W_sl_. The results of shear bond strength on enamel and dentin are shown in Figure 4. The same letters mean that there are no significant differences in that graph. The shear bond stress to enamel of Clearfil SE was significantly higher than others.

**Figure 5:**
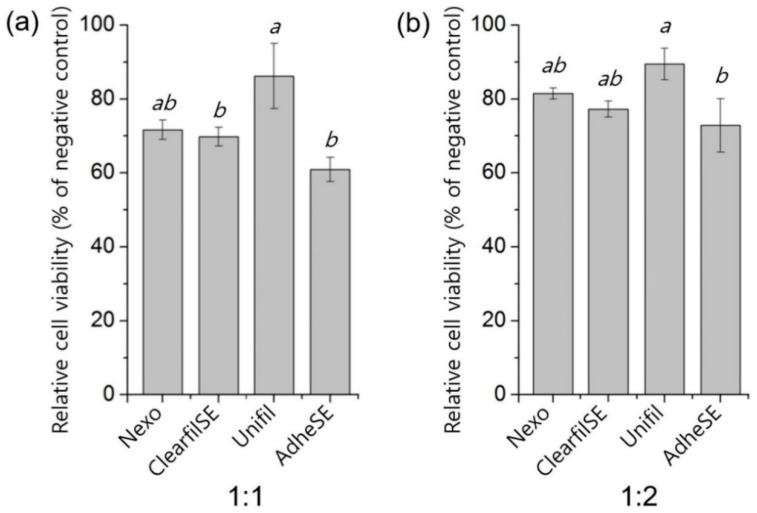
MTT assay (% of negative control). The ratio (**a**) 1:1 means 100% of extraction and (**b**) 1:2 means extraction and non-serum media ratio. The same letters mean no significant differences. Raw data are presented in Tables S6 and S7.

To make sure there is no problem in the cytotoxicity of each group, the MTT assay was performed according to ISO 10993-5 [11]. The results of the MTT assay are shown in Figure 5. The ratio in blanks, 1:1 means 100% of extraction, and 1:2 means extraction and non-serum media ratio. The MTT assay of all groups was shown to be over 60% of relative cell viability in 1:1 except AdheSE.

## 4. Discussion

Dentin bonding is a key in many restorative clinical procedures and is fundamental in aesthetic dentistry. The basic mechanism of dentin adhesive is the resin bonding tag in dentin holes that connects the dentin and resin composites. To satisfy this mechanism, various dentin adhesives have been developed for more than half a century [13].

The total etching system was the only method; however, acidic functional monomer is incorporated into dental adhesive. The functional monomers play an essential role on the bonding and physicochemical properties. The mechanism of functional monomer, such as MDP, is calcium in enamel and dentin chemically bonded and the MDP-Ca salts on the dental hard tissue. In detail, it is known that when MDP-containing adhesives come into contact with dentin, the abutting surfaces are partially demineralized to a submicron depth. Due to this reaction, calcium ions released upon partial dissolution of hydroxyapatite (HAp) diffuse in the hybrid layer. These diffused calcium ions influence the assembly of MDP molecules into nanolayers driven by MDP-Ca salt formation. In conclusion, the chemical structure of 10-MDP monomer allows for favorable polar behavior for adhesion and is known to promote the protection of collagen fibers through MDP-calcium salts formation [14]. Therefore, they create a particular nano-layered structure with the remaining dentin HAp [3,15].

The dentin adhesives require proper characters in the clinic, and among the various characters of a dentin adhesive, a bonding strength to enamel and dentin, a water sorption and solubility, and biocompatibility are considered important properties. However, few standards were applicable for the test dentin adhesives. In this study, we evaluated the materials according to ISO 4049 polymer-based dental materials [8].

After releasing the patent, MDP has been modified in many self-etching adhesives, and we also added the MDP for a self-etching system. The synthesized monomer was confirmed by ^1^H NMR, and hydrogen was detected at the same peaks. Commonly used acid monomers in dental adhesives are usually constituted in an acidic group, a polymerizable group, and a spacer, like an aliphatic chain [16]. In addition, MDP and N,N′-diethyl-1,3-bis(acrylamido)propane (DEBAAP) have been mainly used in a self-etching system. To evaluate and compare new products, we purchased three products which were classified as same generation. Only ClearfilSE contained MDP, and the new Nexo was comparable in the aspect of monomer. AdheSE contained acidic monomer, DEBAAP, which could show high mechanical properties with low quantity [16]. The Unifil contains 4-methacryloxyethyl trimellitic acid (4-MET) with ethanol or acetone; however, it was reported that methacryloxy ester portion in the 4-MET resulted in the hydrolysis reaction [17].

We measured the film thickness according to ISO 4049, one of the important properties of resin-based cements [8]. Compared to the resin cement, the dentin adhesives did not contain filler for strength, but only monomers. Although the values of all test results were compiled, we assumed that other test methods or other standards for its flowability were required.

The water sorption and solubility tests have been conducted on many dental adhesives and, generally, the extent and rate of these properties increased with the hydrophilicity of the resin compositions [18]. Among the monomers, hydroxyethyl methacrylate (HEMA) is well known as a hydrophilic monomer. Malacarne et al. reported that lower rates of water uptake showed less hydrophilic adhesive [18]. All test groups contained HEMA in primer and bonding, and the Nexo group showed the lowest values in both results. The water solubility was under 7.5 μg/mm^3^ which is an acceptable requirement in ISO 4049 [8]. On the other hand, of all data, the water sorption was higher than its requirement. Yoshida et al. reported that the addition of the HEMA also influenced not only water sorption, but also the chemical bonding of MDP by inhibited interfacial nano-layering [19], but we could not resolve its character with bonding strength related to MDP and HEMA in this study.

Common etchants are not as effective as phosphoric acid in enamel bond strength [20]. As a result, in the process of enamel exposure, self-etching adhesives cannot guarantee a satisfactory shear bond strength value for dental restorations for bonding orthodontic brackets [21]. The porous hybrid layer formed in this process has a sub-micron thickness, and in this structure, the micromechanical retention provided by the interlocked resin monomer is the principle mechanism of adhesion [1]. The most important characteristic is bonding strength to enamel or/and dentin. Based on the improvement of these problems and the physical/chemical theories, test evaluations of materials used in dentistry are also ongoing, and through this, problems are steadily improved and new theories are emerging [21]. In this study, we conducted a shear bond strength test according to ISO 29022 [9]. There were three kinds of bond strength, and among them, we conducted a notched-shear bond strength test. All high values of shear bond strength required various factors such as tooth surface, adhesion surface area, parallel specimen, and composite resin used in the study. Therefore, the results are not comparable with previous tests. In this study, the enamel and dentin shear bond strength in the same groups were similar, and the Nexo group showed no significantly different result to Clearfil SE. Even though we conducted tests on at least fifteen teeth according to the test method, the range of standard deviation was huge in enamel bonding strength. Previous enamel etching resulted in fewer marginal defects and marginal discoloration, compared with using the Clearfil SE approach alone. For restoration retention, the differences between the two groups were not significant [22]. In the previous study, they mentioned that self-etching showed promising results in superficial and deep dentin [23], and when properly handled, self-etching is more effective to dentin than total etching [24,25].

Understanding the mechanism of cytotoxicity is necessary for selection of a strategy to protect the dentine-pulp complex. In the ISO 7405 for biocompatibility of dental materials [26], there is a special test method for dentin-pulp structure connective applications. Schmalz et al. and Ulker et al. conducted the test in dentin barrier cell culture structure. They reported that this test method was very similar to the in vivo situation, where the material covers the dentinal walls and only indirectly interacts with the pulp, by means of the tubular fluid [27,28,29]. The cytotoxicity of dentin adhesives was affected by its ingredients, especially uncured monomers [30]. In this study, the cytotoxicity test was conducted only with a bonding agent due to its characteristics. The primer contains acidic monomer, and the product showed pH lower 2.0. In this study, the limitation was that we just evaluated the cytotoxicity of the adhesive, which showed mild cytotoxicity. In addition, cytotoxicity tests using extractions of dental adhesives were a more severe method than the commonly used method by indirect contact dentin barrier.

In this study, the hypothesis of this paper was rejected, and the new blended two-bottle self-etching adhesive system was comparable to the products in the market. Although the various dentin adhesive systems have been developed and evaluated in previous research, more research is needed to prove their properties in vitro and in vivo. Moreover, more long-term clinical case reports are needed.

To approve the product on the market, more experiments need to be conducted. In addition, selective enamel etching and scrubbing techniques should continue to be developed in order to improve penetration and solve the problem of stable bonding [14]. Compared to the development of generation, the guideline may not offer full coverage. Appraisal criteria should be developed by reputable organizations such as the International Organization for Standardization (ISO) to guide manufacturers, or scientists who are interested in patenting their own inventions. Finally, the cautious forecasting of domestic production in self-etching adhesive is expected.

## 5. Conclusions

The newly-developed two-bottle self-etching adhesive containing MDP showed significant results compared to other products on the market and more formal research (case reports and case series) is needed to promote its production for the market. In addition, the appraisal criteria for dental adhesives need to be developed.

## Figures and Tables

**Table 1:** The SMILE (Simplified Molecular-Input Line-Entry system) of organic compound with associated CAS (Chemical Abstracts Service) number.

Compound Name	10-Methacryloyloxydecyl Dihydrogen Phosphate
SMILE	C=C(C)C(=O)OCCCCCCCCCCOP(=O)(O)O
CAS number	85590-00-7

## Supplementary Materials

The following are available online at https://www.mdpi.com/1996-1944/13/16/3553/s1, Table S1: Thickness of bonding agent (μm). The same letters mean no significant differences, Table S2: Water sorption (μg/mm^3^). The same letters mean no significant differences, Table S3: Water solubility (μg/mm^3^). The same letters mean no significant differences, Table S4: Shear-bond strength to enamel (MPa). The same letters mean no significant differences, Table S5: Shear-bond strength to dentin (MPa). The same letters mean no significant differences, Table S6: MTT assay (% of negative control). The same letters mean no significant differences, Table S7: MTT assay (% of negative control). The same letters mean no significant differences.
