# Supplementary material for: Characteristics of 10-Methacryloyloxidecyl Dihydrogen Phosphate Monomer in Self-Etching Two-Bottled Dental Adhesive System: Comparison with Commercial Products
Source: Materials (Basel). 2020 Aug 12;13(16):3553. doi: 10.3390/ma13163553 (PMC7475973; doi:10.3390/ma13163553)
Supplement: Supplementary file 1 [file materials-13-03553-s001.pdf]

*materials*

*Supporting Materials*

# Characteristics of 10-Methacryloyloxidecyl Dihydrogen Phosphate Monomer in Self-Etching Two-Bottled Dental Adhesive System: Comparison with Commercial Products

**Jiyeon Roh, Hyunjung Shin and Min-Ho Hong**

**Table S1.** Thickness of bonding agent (μm). The same letters mean no significant differences.

| Nexo | Clearfil | Unifil | AdheSE |
|---|---|---|---|
| 5.0 ± 1.4 [b] | 6.5 ± 2.5 [ab] | 5.5 ± 2.8 [b] | 8.9 ± 2.5 [a] |

**Table S2.** Water sorption (μg/mm³). The same letters mean no significant differences.

| Nexo | Clearfil | Unifil | AdheSE |
|---|---|---|---|
| 67.40 ± 1.96 [d] | 78.94 ± 3.32 [c] | 85.18 ± 4.56 [b] | 95.37 ± 0.64 [a] |

**Table S3.** Water solubility (μg/mm³). The same letters mean no significant differences.

| Nexo | Clearfil | Unifil | AdheSE |
|---|---|---|---|
| 5.83 ± 0.49 [c] | 12.71 ± 0.46 [a] | 8.74 ± 0.64 [b] | 9.53 ± 1.14 [b] |

**Table S4.** Shear-bond strength to enamel (MPa). The same letters mean no significant differences.

| Nexo | Clearfil | Unifil | AdheSE |
|---|---|---|---|
| 20.66 ± 4.81 [ab] | 24.30 ± 7.56 [a] | 18.20 ± 5.96 [bc] | 12.53 ± 6.34 [c] |

**Table S5.** Shear-bond strength to dentin (MPa). The same letters mean no significant differences.

| Nexo | Clearfil | Unifil | AdheSE |
|---|---|---|---|
| 17.22 ± 5.50 [a] | 22.40 ± 9.19 [a] | 22.14 ± 7.34 [a] | 19.51 ± 7.38 [a] |

**Table S6.** MTT assay (% of negative control). The same letters mean no significant differences.

| Nexo | Clearfil | Unifil | AdheSE |
|---|---|---|---|
| 71.66 ± 2.58 [ab] | 69.78 ± 2.49 [b] | 86.19 ± 8.79 [a] | 60.89 ± 3.27 [b] |

100% of extraction from specimen.

**Table S7.** MTT assay (% of negative control). The same letters mean no significant differences.

| Nexo | Clearfil | Unifil | AdheSE |
|---|---|---|---|
| 81.49 ± 1.50 [ab] | 77.25 ± 2.15 [ab] | 89.46 ± 4.26 [a] | 72.82 ± 7.26 [b] |

Extraction and non-serum media ratio is 1:2.

© 2020 by the authors. Submitted for possible open access publication under the terms and conditions of the Creative Commons Attribution (CC BY) license (http://creativecommons.org/licenses/by/4.0/).
